# Arthroscopic surgery versus physiotherapy for femoroacetabular impingement: a meta-analysis study

**DOI:** 10.1007/s00590-020-02675-6

**Published:** 2020-05-07

**Authors:** Matthias Gatz, Arne Driessen, Jörg Eschweiler, Markus Tingart, Filippo Migliorini

**Affiliations:** Department of Orthopaedic Surgery, RWTH Aachen University Clinic, Pauwelsstraße 30, 52074 Aachen, Germany

**Keywords:** Femoroacetabular impingement, FAI, Treatment, Arthroscopy, Conservative, Physiotherapy

## Abstract

**Introduction:**

Femoroacetabular impingement (FAI) is thought to play an important role in the development of hip osteoarthritis. However, there is no consensus about the optimal treatment options, since non-operative therapy such as physiotherapy and surgical treatment such as arthroscopic hip surgery can both improve symptoms. Therefore, the aim of the present meta-analysis was to compare the outcomes between two different treatment regimes; physiotherapy versus arthroscopic treatment for FAI.

**Methods:**

The present meta-analysis was carried out according to the PRISMA guidelines. In November 2019, the main online databases were accessed. All the randomized clinical trials (RCTs) comparing surgical arthroscopic treatment versus physiotherapy for FAI were considered for inclusion. Only articles reporting quantitative data under the outcomes of interest were included. For the all analysis, we used Review Manager Software. Data from 644 patients were analysed.

**Results:**

Data from 644 patients were evaluated with a mean follow-up of 14.67 ± 8.3 months. The unpaired *t* test detected an optimal baseline comparability in terms of side, gender, years, duration of symptoms and BMI (*p* = 0.08–0.9). The VAS subscale of the score EQ-5D and the mean iHOT33 reported favourable values in the arthroscopic group (*p* = 0.03 and *p* < 0.0001, respectively). Similar findings were evidenced in the iHOT33 subgroup 6-months (*p* = 0.70) and 12-months (*p* = 0.0002). The HOS score, the ADL (*p* < 0.0001) and the sport (*p* = 0.0003) subscales reported both greater values in the arthroscopic group. No statistical significance was found concerning the risk to incur in further total hip arthroplasty (*p* = 0.72).

**Conclusion:**

Based on only three high-quality RCTs, arthroscopic hip surgery is an effective therapeutic treatment for FAI revealing superior results than a non-surgical approach with physiotherapy.

## Introduction

Femoroacetabular impingement (FAI) is thought to be responsible for up to 50% [1] of all hip osteoarthritis. FAI is based on either a cam morphology which describes a loss of sphericity of the femoral head or on a pincer morphology which describes an exuberant acetabular coverage of the femoral head or a combination of both. Moreover, both morphologies might occur in combination. FAI morphology can be found in up to 20% of the general population [2], but only one-fourth develop symptoms or osteoarthritis [3]. Consequently, it is of great importance to screen predominantly young patients with actual or future symptoms providing the best therapy in order to avoid or at least postpone end stage osteoarthritis requiring hip arthroplasty.

Among others, two main treatment pathways exist: the traditional conservative approach that takes advantages of specific physiotherapy protocols and the arthroscopic surgery that is receiving always more consensus. Proponents of conservative treatment argue that not all individuals with radiographic FAI signs develop symptoms and therefore correcting the deviating anatomical structure may not address the underlying pathology comprehensively, since hip muscle weakness, lower trunk strength and poor dynamic single-leg balance and further dysfunctional muscular impairments cannot be treated surgically [4]. Physiotherapy is based on patient education and advice, patient assessment, help with pain-relief and an exercise-based hip programme with stretching and strengthening avoiding painful hard end stretches [5]. Contrary to the conservative procedure, advocates of the arthroscopic surgery approach address the anatomical impairments by reshaping the hip and/or the acetabulum, since especially in flexion and internal rotation premature contact between the femoral head-neck junction and the anterior rim of the acetabulum causes painful labrum and cartilage degeneration and thus possible promoting osteoarthritis [6]. Despite the fact that, e.g. in England numbers of hip arthroscopy increased by over 700% between 2002 and 2013 [7], there is an immense lack of evidence giving the impression that “FAI surgery has evolved rapidly and at a pace far quicker than our understanding about the natural history and epidemiological characteristics of the condition” [6]. In 2014, a Cochrane systematic review could not made sufficient conclusions due to the lack of available studies only observing three ongoing studies comparing hip arthroscopy versus non-operative treatment [6]. However, it is in the nature of things that surgical treatment implies higher risks and fatal harms and therefore providing explicit treatment recommendation is of great importance for both patients and physicians a guided choice in the treatment of FAI. Consequently, to provide data for evidenced-based decision making in the treatment for FAI, a comprehensive meta-analysis study comparing the outcomes of physiotherapy versus arthroscopic hip surgery was conducted.

## Materials and methods

### Search strategy

The present meta-analysis was carried out according to the Preferred Reporting Items for Systematic Review and Meta-Analysis: the PRISMA statement [8]. The search strategy was guided as the follows:

- (P) Population: FAI;
- (I) Intervention: arthroscopic treatment;
- (C) Comparison: conservative treatment;
- (O) Outcomes: clinical and functional scores, progression to OA and THA.

### Literature search

Two independent authors (MG, FM) performed the literature search. In November 2019, the main online databases were accessed: Pubmed, Embase, Google Scholar, Scopus. The following keywords were used in combination: *femoroacetabular impingement, FAI, pincer, cam, arthroscopy, conservative, physiotherapy, versus*. If title and abstract matched the topic, the full-text article was accessed. The bibliography of the included papers was also screened. Disagreements between the authors were mutually debated and solved.

### Eligibility criteria

All randomized clinical trials (RCT) comparing arthroscopic versus conservative treatment for FAI were considered for inclusion. According to the Oxford Centre of Evidenced-Based Medicine [9], only articles level one of evidence were included in the present study. There was no restriction to the year of publication. According to the authors language capabilities, articles in English, Italian, German, Spanish, Portuguese and French were considered for inclusion. Articles dealing with open surgery or arthroscopic surgery in existing osteoarthritis were excluded. Only articles reporting quantitative data under the outcomes of interest were included in the present study. If data under the outcomes of interest were missing, the authors were contacted to provide missing data and if positive the studies were included.

### Outcomes of interest

Two independent authors (MG, FM) performed data extraction. The following demographic data were collected: author, year and journal, duration of the follow-up, number of procedures, side (%), gender (%), duration of the symptoms (months), mean age and Body Mass Index. Moreover, according to data availability, the following outcomes of interest were extracted: iHOT33 score and subscale 6 and 12 months, the subscales ADL and sport of the HOS score, the VAS subscale of the score EQ-5D, the risk of incur in further total hip arthroplasty.

### Methodological quality assessment

For the methodological quality assessment, we referred to the risk of bias tool of the Review Manager Software version 5.3 (The Cochrane Collaboration, Copenhagen). The following risk of publication bias was analysed: selection, detection, attrition, reporting and unknown. The risk of bias was performed by two independent authors (MG, FM).

### Statistical analysis

The statistical analysis was performed by one author (FM). The IBM SPSS 24.0 was used to assess baseline comparability, with values of the unpaired *t* test > 0.5 considered to be satisfactory. For the meta-analyses, the Review Manager Software version 5.3 (The Cochrane Collaboration, Copenhagen) was used. For continuous variable, the estimated effect was evaluated through the inverse variance statistical method, using the mean difference (MD) effect measure. Dichotomous variables were analysed through the Mantel–Haenszel statistical method using the odd ratio (OR) effect measure. To evaluate the risk of publication bias, the funnel plot was performed. This evaluated the final effect by plotting the mean difference of the most reported endpoint against the standard error, SE(MD). Heterogeneity was evaluated through the *χ*^2^ and Higgins-*I*^2^ tests. If *χ*^2^ > 0.5 and *I*^2^ > 60% heterogeneity affected considerably the results. A fixed model effect was set in all the comparisons. In event of high heterogeneity, a random model effect was used. The confidence interval (CI) was set at 95% in all the comparisons. Values of *p* < 0.05 were considered statistically significant.

## Results

### Search result

**Fig. 1:**
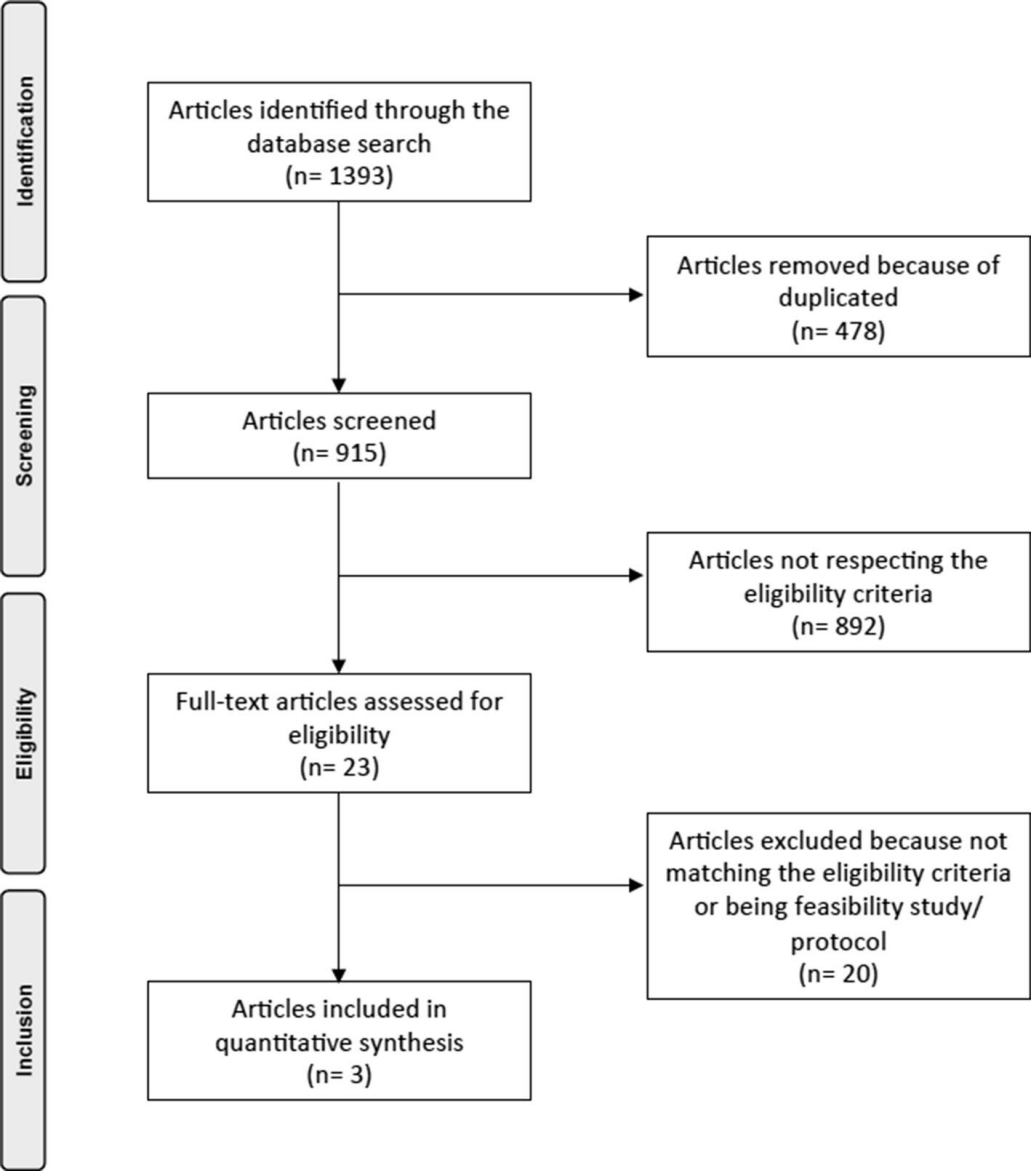
Flow chart of the literature search

The literature search resulted in 1393 papers with 915 articles screened for inclusion after removing duplicates (*n *= 478). A total of 892 papers were excluded due to incompatibility with the eligibility criteria. Further, 20 articles were excluded because of being feasibility studies or study protocols or not matching the eligibility criteria. This last operation left 3 RCTs for the present study. The flow chart of the literature search is shown in Fig. 1.

### Methodological quality assessment

**Fig. 2:**
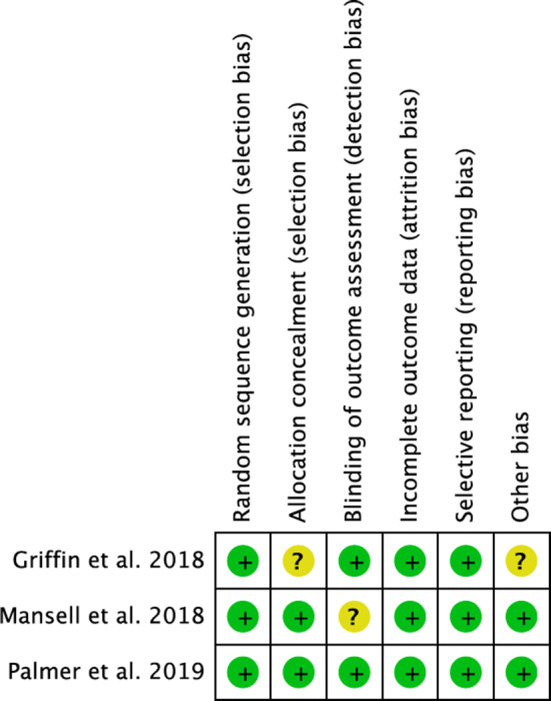
Cochrane Collaboration risk of bias summary

Cochrane risk of bias tool detected a low risk of selection, detection, attrition and reporting bias. All the included articles were of high-quality, providing randomization and blinding of assessor or a physician, with an optimal data analysis and results interpretation. Concluding, the methodological quality assessment of the present meta-analysis was outstanding. The risk of bias according to each study is shown in Fig. 2.

### Risk of publication bias

**Fig. 3:**
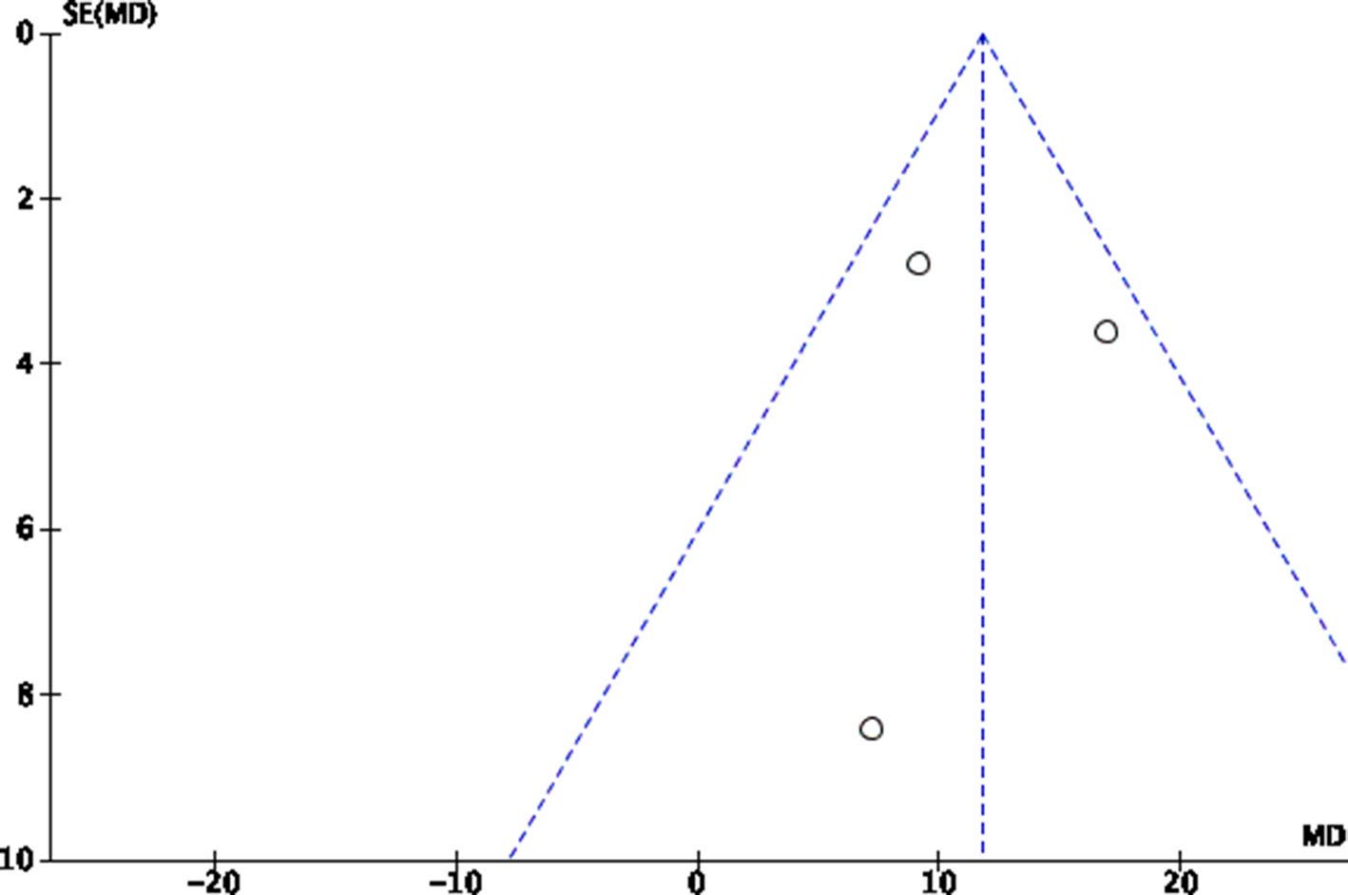
Funnel plot of the most reported endpoint (iHOT33)

The risk of publication bias was evaluated through the funnel plot of the most reported endpoint (iHOT33). The plot detected a quite symmetry between the referral points. No values were evidenced outside the range of acceptability, close to the no-effect line. Concluding, the risk of publication bias was acceptable (Fig. 3).

### Patient demographic

Data from 644 patients were collected. The mean follow-up was 14.67 ± 8.3 months. In the arthroscopy group, a total of 346 patients were analysed: 58% right side, 66% female gender. The mean duration of the symptoms was 29.5 ± 10.6 months, the mean age was 33.83 ± 3.6 years, the mean BMI was 27.01 ± 1.6 kg/m^2^. In the physiotherapy group, a total of 298 patients were analysed: 56% right side, 46% female gender. The mean duration of the symptoms was 30.5 ± 13.4 months, the mean age was 33.75 ± 3.2 years, the mean BMI was 27.03 ± 0.6 kg/m^2^. The unpaired *t* test found no differences concerning side (*p* = 0.3), gender (*p* = 0.08), symptoms duration (*p* = 0.5), age (*p* = 0.9) and BMI (*p* = 0.9). The patient demographic is shown in Table 1.

**Table 1:** Demographic assessment of the included studies

Author, year, (study title)	Journal	Type of deformity	Inclusion criteria	Exclusion criteria	Mean follow-up (months)	Intervention	Procedures (*n*)	Previous symptoms duration (months)	Right: left (*%*)	Female (*%*)	Mean age	Mean BMI	Surgical technique	Rehabilitation program
Mansell et al. 2018 [10]	*Am J Sports Med*		(1) Age 18–60 (2) positive flexion adduction internal rotation test (3) relief after intraarticular injection (4) joint space > 2 mm (5) positive crossover sign (6) alpha angle > 50° (6) failed 6 weeks of conservative management	(1) Osteoarthritis (2) systemic disease (3) formal course of physiotherapy within last 6 months	24	Arthroscopy	63	41 ± 62	61:39	40.9	30.3	27.7	(1) Acetabuloplasty (2) labral repair/debridement (3) femoroplasty	
						Conservative	11	24 ± 142	57:43	42.9	29.4	32.9		(1) 12 sessions with a physical therapist (2) personalised impairment-based treatment plan (3) joint mobilisations, mobilisation with motion, therapeutic exercise, soft tissue mobility, stretching and motor control exercises to address the identified impairments (4) additional home training
Griffin et al. 2018 [11] (UK FASHIoN)	*The Lancet*	Pincer (27) Cam (262) Mixed (59)	(1) Age > 16 (2) hip pain (3) alpha angle > 55° (4) lateral centre-edge angle > 40° (5) positive crossover sign (6) > 2 mm joint space	(1) Osteoarthritis (2) congenital/adolescent hip diseases (3) fracture	12	Arthroscopy	171	37 ± 36	56:44	42	35.4		(1) Acetabuloplasty (2) labral repair/debridement (3) femoroplasty	Physiotherapy routine care distincted from the study, improving ROM and return to activity
						Conservative	177	40 ± 40	58:42	36	35.2			(1) 6–10 sessions over 12–24 weeks with physiotherapist personalised hip therapy with assessment of function, pain and range of motion with individualisation, progression and supervision
Palmer et al. 2019 [12] (FAIT)	*BMJ*	Pincer (1) Cam (208) Mixed (13)	(1) Age 18–60 (2) hip pain with non-defined imaging parameters for FAI	(1) Osteoarthritis (2) formal course of physiotherapy within last 12 months (3) hip dysplasia (4) previous surgery	8	Arthroscopy	112		60:40	66	36.4	25.9	(1) Acetabuloplasty (2) labral repair/debridement (3) femoroplasty (4) microfracture	Physiotherapy routine care distincted from the study, improving ROM and return to activity
						Conservative	110		54:46	66	36.0	26.6		(1) Up to 8 physiotherapy sessions during 20 weeks with physiotherapist personalised hip therapy, with emphasis on improving core stability and movement control

### Outcomes of interest

**Fig. 4:**
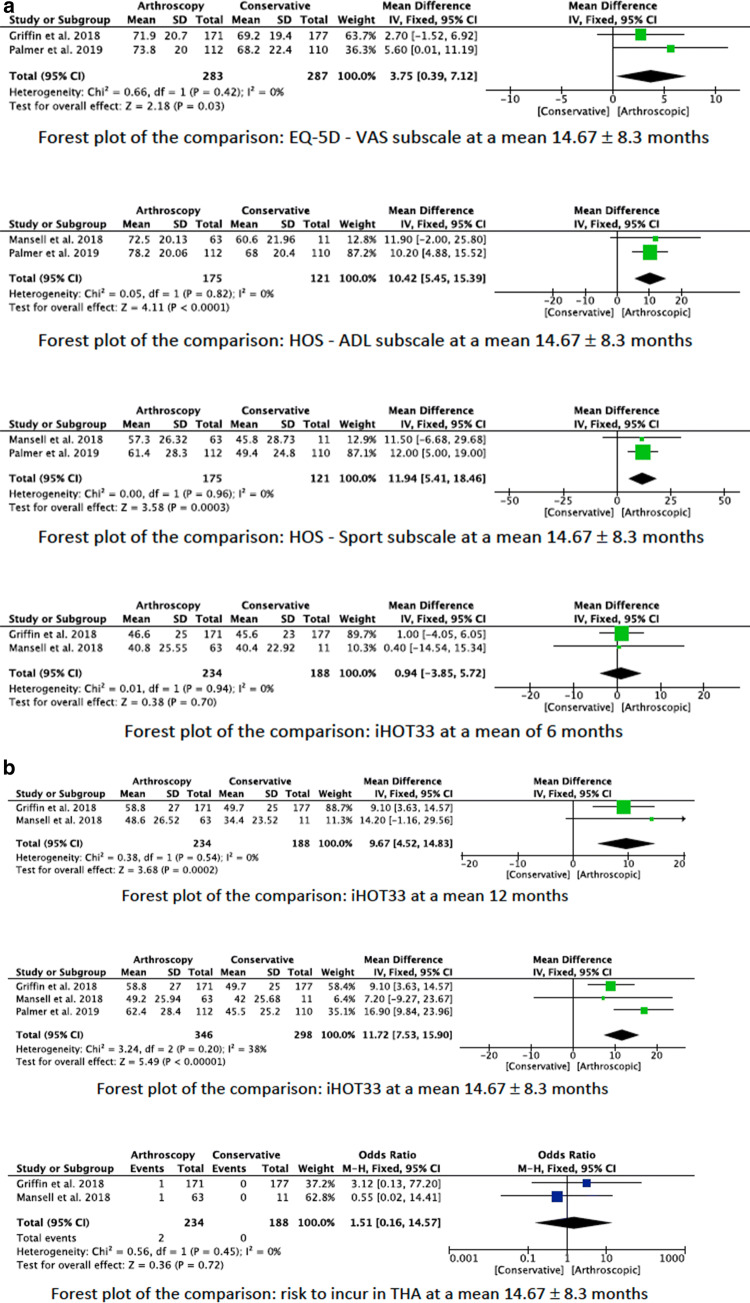
Overall comparisons

The mean iHOT33 showed greater values in favour of the arthroscopic surgery group (EE 11.72; 95% CI 7.53–15.90; *p* < 0.0001). Similar findings were evidenced in the iHOT33 subgroup 6-months (EE 0.94; 95% CI − 3.85 to 5.72; *p* = 0.70) and 12-months (EE 9.67; 95% CI 4.52–14.83; *p* = 0.0002) follow-up. Concerning the HOS score, the ADL (EE 10.42; 95% CI 5.45–15.39; *p* < 0.0001) and the sport (EE 11.94; 95% CI 5.41–18.46; *p* = 0.0003) subscales reported both greater values in the arthroscopic surgery group. The VAS subscale of the score EQ-5D reported favourable values in the arthroscopic surgery group (EE 3.75; 95% CI 0.39–7.12; *p* = 0.03). Reduced rate of OA progression (OR 0.23; 95% CI 0.05–1.15) and rate of THA (OR 1.51; 95% CI 0.16–14.57) were evidenced in the arthroscopic group. However, they were not significant (*p* = 0.07 and *p* = 0.7, respectively). The forest plots of the comparisons are shown in Fig. 4.

## Discussion

According to the main findings of the present meta-analysis, arthroscopic surgery revealed higher outcome values in all examined scores (iHOT33; HOS-ADL; HOS-sport; EQ-5D) than a conservative physiotherapy-based treatment regime for FAI. The iHOT33 is a 33-item questionnaire especially for young active persons with hip problems covering in total four sections such as symptoms and functional limitations, sports and recreational activities, known as a reliable tool to quantify symptom changes [13]. The iHOT33 showed significantly (*p* < 0.0001) greater mean values in favour of the surgical treatment group. Furthermore, the arthroscopic surgery group revealed greater values in the HOS score being an established outcome tool with clinometric evidence [14] in the subscales ADL (*p* < 0.0001) and sport (*p* = 0.0003). Additionally, favourable results could be detected for quality of life and pain assessment as well as psychological factors using EQ-5D [15] (*p* = 0.03) for the arthroscopic group.

### Arthroscopic surgery

There are several factors contributing to the overall better results in the arthroscopic surgery group. First of all, the surgical procedure itself by correcting the biomechanical impairments with reshaping bony structures and labrum repair might reveal positive outcome measures. Nevertheless, it has to be taken into account that due to the nature of the included studies surgical treatment might have a significant placebo effect. To investigate this influence there are at least two on—going clinical trials comparing arthroscopic osteochondroplasty with arthroscopic lavage (FIRST trial) or with sham surgery (HIPARTI trial) [16, 17]. Moreover, it has to be taken into account that also the surgically treated individuals received a post-operative physiotherapy where functional impairments might have been additionally treated. The FAIR trial starting in 2013 tried to find evidence concerning this issue, but unfortunately only revealed limited results due to recruitment difficulties and funding constraints. Only 30 participants were included having superior results with a post-operative rehabilitation programme at 14 weeks but not at 24 weeks in comparison to a control group without post-operative rehabilitation [18]. To sum up, it remains unclear which of the factors—the surgical treatment itself, placebo effect or post-operative rehabilitation—was crucial [11]. However, the combination of all displays a real-world setting and will be hardly separated from each other, since studies about sham surgery or waived post-operative rehabilitation are challenging.

### Physiotherapy

Physiotherapy is thought to improve both pain and function by activating muscle strengthening and stabilisation patterns and by reducing unfavourable movements leading to an painful impingement syndrome [11]. However, in their editorial note in the BJSM (2019) Kramp et al. pointed out, that the results in the included studies might be questionable if the type, dose and duration of the physiotherapy reported in the trials (FAIT: 8 sessions; FASHIoN: 10 sessions; Mansell et al.: 12 sessions) have been sufficient and that the physiotherapy treatment protocol might not be considered as the current best practice anymore [4, 10, 11]. Nevertheless, Griffin et al. [5] tried to give best conservative care based on clinical experience and the given possibilities within the UK National Health Service with the development of the “personal hip therapy” protocol. However, the protocol also included cortisone injections as an additional module [5]. Consequently, several studies such as “PhysioFirst” are needed to provide further validated evidence for physiotherapy especially in the case of a non-operative treatment pathway as well as in the perioperative setting [4].

### Strengths and limitations

The most important point of strength of the present study is represented by the high quality of the methodological assessment. All the studies provided randomization, blinding score assessment methods and were based on previously published protocols or preliminary studies. Moreover, the FAIT and FASHiON trials are large multi-centre trials emphasizing the generalizability of the results [11, 19]. These characteristics correlate with low risk of selection and detection bias, ensuring reliable and trustworthy results having.

The results of the present meta-analysis have to be interpreted in the light of the following limitations. The most relevant limitation of the present study is the reduced number of papers eligible for inclusion and overall procedures. Up to date, the only further registered RCT studies in the International Clinical Trial Register of the WHO comparing arthroscopic versus physiotherapy is the Australian FASHioN trial, but data has not been published yet [12]. Therefore, only limited high-quality data can be expected in the next years, but hopefully further RCT studies will be designed to improve data pooling. Having a small study sample, specific limitations of each single study influence the results of the present meta-analysis: The study by Mansell et al. [10] was a single-centre study with one surgeon having a high rate of crossover influencing the power and making a type II error possible. In the studies by Griffin et al. and Palmer et al., score evaluation was set after randomization, but there was a frequent delay in delivery of surgery, so that the arthroscopic group had in general a reduced recovery time [11].

A further relevant limitation of this meta-analysis is the relatively short follow-up period. Only the study by Mansell et al. evaluated the outcome after 24 months, while the FAIT and UK FASHIoN trial had a follow-up of only 8 months and 12 months, respectively. This limits clearly the evaluation of long-term outcome parameters like the prevention of hip arthroplasty. Moreover, no analysis of the various impingement morphologies was possible, because CAM-Impingement was the predominant type in the analysed studies with only limited cases of Pincer and mixed FAI.

A further significantly considered limitation is the incongruence between clinical score improvement and general subjective changes, reducing the explanatory power of clinical results. Even though the iHOT-33 and HOS score are validated scores for FAI, the minimal clinically important difference (MCID) does not seem to directly correlate to the subjective improvement of the patients. Even though Mansell et al. reported a score improvement surpassing the MCID of the HOS sport subscale and the iHOT33 only a minority was totally satisfied (Mansell: 45.2%; Palmer et al. (FAIT): 51%) [10, 19]. The Forest plots in Fig. 4 show that in the present meta-analysis the MCID between arthroscopic surgery and physiotherapy are reached for all measurement points of the iHOT33 and HOS score except after 6 months for the iHOT33. However, none of the included RCT’s analysed the Fragility index for studying the robustness of given data [20].

We were not evaluating the complications of surgery versus physiotherapy, since it is in the nature of things that a surgical procedure has a higher rate of side effects. In this sense, it is mandatory for future studies to justify the use of arthroscopic surgery with a better outcome. However, the available data considered to fewer cases to give a validated conclusion, since the reported rate of severe complications is already very low in arthroscopic surgery [21, 22]. In all surgical procedures (*n *= 346) of the included studies, there were only *n *= 2 severe complications (*n *= 1 fracture, *n *= 1 hip infection).

A further limitation is that, none of the included studies investigated “return to sports”, which is a crucial factor in the rehabilitation of the predominantly young cohort suffering from FAI. However, Mansell et al. [10] investigated military patients and stated that about 50% could return to active military work without significant differences between the cohorts. Nevertheless, further research is mandatory, as participation, load and performance in sports is still remarkably reduced 1 year after arthroscopic surgery [4].

Moreover, further limiting factors are the various operative and physiotherapeutic treatment differences within and between the three studies, being a clear confounding factor. Particularly, for the surgical procedures therapeutic variations like capsular closure versus non-closure or labral repair versus labral debridement might influence clinical findings.

### Clinical implications and future directions

Taking the current literature into account, the intention of this meta-analysis was to give an evidence-based recommendation about the efficacy of an arthroscopic intervention in FAI. Therefore, based on the results of this meta-analysis one can clearly invalidate the apprehension of the Cochrane Review of 2014 that arthroscopic surgery does not have any evidence-based status in the treatment of FAI despite its widen usage [6]. Contrary, the present meta-analysis gives a sufficient evidence, that an arthroscopic procedure is a successful therapeutic option, although it is too early to pronounce it as the number one treatment option in FAI. Since current evidence is only limited on three RCTs and physiotherapy is an easy accessible and not harmful alternative, it is assumable that physiotherapy will still play a key role especially as an important component in a perioperative setting. So far, the Warwick Agreement might be helpful to choose the optimal treatment in clinical practice [23]. This international consensus statement recommends a shared decision-making process depending on the individual patient with the triad of symptoms, clinical signs and imaging findings [23]. Nonetheless, a recent systematic review depicted that in daily routine imaging findings showed to be a criterion for surgery in 92%, symptoms in 75% and clinical test in 70% of the studies, whereas only 56% utilised the combination of all three factors [24].

For the future direction in the field of research, studies need to consider the relation between clinical scores and the MCID, patient acceptable symptomatic state (PASS) and the fragility index in order to find out what really contributes to the clinical changes, besides reporting only significant score differences between study cohorts. Additionally, future studies need to reveal the influence of perioperative physiotherapy and the placebo effect of the surgical procedures. There is a need to provide data about the right timing for arthroscopic surgery, since physiotherapy might be still considered as an useful first therapeutic module. In this case, long-term studies are of particular interest considering further factors like the specific type of impingement, the exact anatomic pathological and the conducted surgical procedure. Consequently, future studies need to define clear indications for surgical therapy and to provide data for supporting evidence.

## Conclusion

For FAI, arthroscopic surgery had better overall outcomes compared to a conservative physiotherapy-based treatment. Therefore, arthroscopic surgery is an adequate treatment option for FAI and might be considered as treatment of first choice in selected patients. Nevertheless, only three high-quality RCTs were included in the present meta-analysis, and future studies need to provide further evidence and specify indications for physiotherapy and arthroscopic surgery.

## Data Availability

Not applicable.

## Abbreviations

FAI: Femoroacetabular impingement
RCT: Randomized clinical trials
EE: Estimated effect
OR: Odd ratio
CI: Confidence interval
MD: Mean difference
MCID: Minimal clinically important difference
PASS: Patient acceptable symptomatic state
